# Electronic Outbreak Surveillance in Germany: A First Evaluation for Nosocomial Norovirus Outbreaks

**DOI:** 10.1371/journal.pone.0017341

**Published:** 2011-03-08

**Authors:** Anja M. Hauri, Hans-Jürgen Westbrock, Herman Claus, Steffen Geis, Siegfried Giernat, Michael Forβbohm, Helmut Uphoff

**Affiliations:** 1 Hesse State Health Office, Dillenburg, Germany; 2 Robert Koch-Institut, Berlin, Germany; 3 Postgraduate Training in Applied Epidemiology (PAE), Robert Koch Institute, Berlin, Germany; 4 Public Health Authority, Main-Kinzig Kreis, Gelnhausen, Germany; 5 Public Health Authority, Wiesbaden, Germany; University of Hong Kong, Hong Kong

## Abstract

**Background:**

In Germany, surveillance for infectious disease outbreaks is integrated into an electronic surveillance system. For 2007, the national surveillance database contains case-based information on 201,224 norovirus cases, three-quarters of which are linked to outbreaks. We evaluated the data quality of the national database in reflecting nosocomial norovirus outbreak (NNO) data available in 19 Hessian local public health authorities (LPHAs) and the influence of differences between LPHA's follow-up procedures for laboratory notifications of Norovirus positive stool samples on outbreak underascertainment.

**Methods:**

Data on NNO beginning in 2007 and notified to the 19 LPHAs were extracted from the national database, investigated regarding internal validity and compared to data collected from LPHAs for a study on NNO control. LPHAs were questioned whether they routinely contacted all persons for whom a laboratory diagnosis of norovirus infection was notified. The number of outbreaks per 1,000 hospital beds and the number of cases within NNOs for acute care and rehabilitation hospitals were compared between counties with and without complete follow-up.

**Results:**

The national database contained information on 155 NNOs, including 3,115 cases. Cases were missed in the national database in 58 (37%) of the outbreaks. Information on hospitalisation was incorrect for an estimated 47% of NNO cases. Information on county of infection was incorrect for 24% (199/820) of cases being forwarded between LPHAs for data entry. Reported NNO incidence and number of NNO cases in acute care hospitals was higher in counties with complete follow-up (incidence-rate ratio (IRR) 2.7, 95% CI 1.4–5.7, p-value 0.002 and IRR 2.1, 95% CI 1.9–2.4, p-value 0.001, respectively).

**Conclusions:**

Many NNOs are not notified by hospitals and differences in LPHA procedures have an impact on the number of outbreaks captured in the surveillance system. Forwarding of case-by-case data on Norovirus outbreak cases from the local to the state and national level should not be required.

## Introduction

In 2001, the Protection against Infection Act (Infektionsschutzgesetz: IfSG) standardised the German surveillance system for notifiable diseases [1]. As a result, the national public health institute in Germany, the Robert Koch Institute (RKI), implemented an electronic surveillance system for infectious disease outbreaks in Germany integrated in the case-based electronic surveillance system SurvNet@RKI [2], [3]. Electronic surveillance has become a necessity and many national surveillance systems now rely on or are moving towards electronic reporting systems [4], [5], [6].

With the advent of the IfSG, laboratories have to notify all cases with norovirus positive stool samples to local public health authorities (LPHAs) and physicians have to notify gastroenteritis outbreaks and hospitals nosocomial outbreaks to LPHAs [1]. Illness due to Norovirus infection is generally mild, characterized by acute vomiting and diarrhea, but may be severe and life threatening in risk groups such as elderly and immunocompromised patients [7], [8]. Outbreaks occur in people of all ages and are particularly common in health care settings and residential homes [9]. In Germany, LPHAS use Norovirus notifications to discuss control measures, e.g. with hospitals and to investigate outbreaks. At the local, state and national level reported data are used to describe the epidemiology of norovirus infections. Since 2004, norovirus gastroenteritis has become the most frequently reported disease in Germany [10]. In 2007, 46% (201,224/438,356) of all cases registered in the national database were norovirus cases. Approximately three quarters (74%) of these cases were part of outbreaks [11]. Most outbreaks occurred in hospitals (39%), nursing homes (38%), and day care centres (14%).

Public health surveillance systems should be evaluated periodically to ensure that problems of public health importance are being monitored efficiently and effectively [12]. Many published evaluations have focused on timeliness [13] and completeness of case reports [14], [15], such as for tuberculosis, meningococcal disease or measles. Attention to data quality and representativeness of surveillance systems has been described as insufficient [16]. With the advent of electronic reporting, there is a general temptation to increase the amount of data requested for every case. For example the IfSG requires LPHAs to report case-based information on hospitalisation, fatalities and place of infection [1]. However data on the validity, acceptability and usefulness of this additional reporting of case-based data are still scarce. Furthermore, electronic processing in the current German surveillance system is limited to the transmission of digitized information from the local, to a state and the national level. This implies that the work-load for LPHAs for the collection and manual input of detailed case-based data into the surveillance system may be considerable for diseases with a high reporting incidence. This evaluation examined the accuracy with which the national database reflects nosocomial norovirus outbreak (NNO) data available at LPHAs. Aspects evaluated include: completeness of case-reports, i.e. the amount of cases captured in the national database as an indication of outbreak size, and completeness and validity of data on county of infection and hospitalisation of cases. Working-time required at state level to link outbreak cases was documented.

Undernotification is a well known characteristic of mandatory surveillance systems [14]–[17]. Many LPHAs consider that nosocomial outbreaks are less frequently notified in comparison to laboratory notifications of norovirus positive stool samples. To identify additional not yet notified norovirus outbreaks several Hessian LPHAs strive to contact all persons for whom a laboratory diagnosis of norovirus infection is notified while others limit their follow-up to laboratory notifications suggesting an institutional setting. However, the influence of LPHAs’ following-up of laboratory notifications of norovirus positive stool samples on the number of NNOs ascertained and associated cases has not been investigated previously.

### Reporting procedure

**Figure 1:**
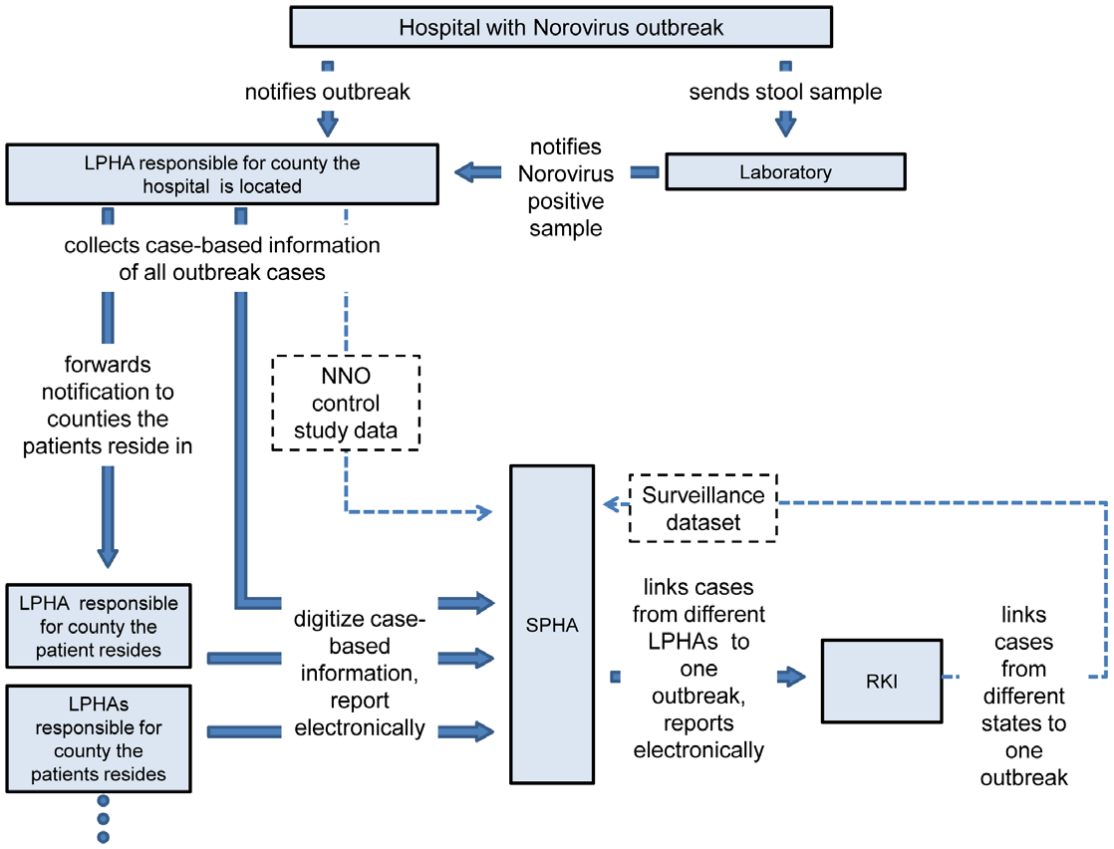
Data flow for the surveillance of nosocomial norovirus outbreaks from hospitals to the national level and – for the evaluation – to the Hessian SPHA, Germany, 2007.

LPHAs use either SurvNet@RKI or one of five commercially offered disease-reporting software systems for the management and transmission of case-based datasets to the state level. The data collected for norovirus cases in this system include demographic characteristics, time of symptom onset and/or diagnosis, hospitalisation, fatalities, presumed place of infection, diagnostics, case definition criteria, association with outbreaks and administrative data. In total, a minimum of 14 items should be entered into the database for each norovirus case. At least once a week the LPHAs are required to enter into the software system newly notified cases fulfilling the national case definition criteria and to create a transport file for the state level (figure 1). Cases are required to be entered into the peripheral database by the LPHA responsible for the county of residence of a case. This implies that LPHAs which investigate a nosocomial norovirus outbreak have to forward information on cases residing outside their counties to the LPHAs where the cases reside, which then enter the case-based data into their databases. State public health authorities (SPHAs) use SurvNet@RKI to store, analyse and forward datasets to the national level at RKI. LPHAs, SPHAs and the RKI can manually link single case records together thereby creating an outbreak report as a new database entity [3]. For this purpose a unique outbreak identification number (OIN), assigned by the LPHA investigating the outbreak is used at local, state and national level. For a case residing outside the county the LPHA investigating the outbreak is responsible for, the OIN has to be forwarded to the LPHA the case resides together with the required case-based information. Linking of cases at local, state and national level is essential to correctly analyse the national database for number and size of outbreaks.

## Methods

Data on NNO beginning in 2007 and notified to 19 of the 25 Hessian LPHAs were extracted from the national database and investigated regarding validity for information on hospitalisation and county of infection. To evaluate the extent to which information on outbreak size available at the LPHAs can be retrieved in the national database, data obtained by these 19 LPHAs during a prospective study on measures taken to control nosocomial norovirus outbreaks, the NNO control study [18], were used.

### Data sources

1) For the NNO control study, 19 of 25 LPHAs prospectively filled in a questionnaire for all outbreaks beginning in 2007 and sent it to the Hessian SPHA at the end of the outbreak (figure 1). Data used from this questionnaire included the OIN and the outbreak size, provided as the number of ill patients and number of ill staff, and is further referred to as the NNO control study dataset.

2) From the national database data on Hessian NNO which began in 2007 were extracted using the following algorithm: First, a list of all Hessian norovirus cases notified in 2007 was used to create a list of all Hessian norovirus outbreaks. Second, all cases which were part of the outbreaks of the Hessian norovirus outbreaks list were extracted from the national database. The second list also contained cases resident outside Hesse and/or notified in 2008. For each single case, information on the setting of the outbreak (e.g. hospital, household, day-care), hospitalisation, place of infection, OIN and the LPHA entering the case into the peripheral database was extracted. Data were extracted from the national database as of 1 July 2008. This dataset was restricted to contain only information on cases whose OIN was included in the NNO control study and is further referred to as the surveillance dataset.

For the excluded cases, information on the setting of the outbreak (e.g. hospital) was used to check for NNO missed in the NNO control study, but none was found. The NNO control study dataset was used to evaluate the national surveillance database regarding internal consistency for information on hospitalisation and place of infection. To evaluate information on outbreak size, the surveillance dataset and the NNO control study dataset were linked based on the OIN. Reasons for differences in outbreak size between the two datasets were determined in collaboration between the 19 LPHAs and the Hessian SPHA. Stata version 10.0® (StataCorp., College Station, TX, USA) was used for data management and analysis.

For this evaluation only data required for mandatory surveillance were used. Hence, no ethical approval was obtained.

### Definitions and assumptions

#### Case definition

The national case definition for norovirus gastroenteritis requires a laboratory confirmation of norovirus infection or symptomatic disease (vomiting or diarrhoea) in a person with an epidemiological link to a laboratory confirmed case [19]. Outbreaks contain ≥2 cases [11].

#### Place of infection

For the national surveillance system, place of infection is defined as the German county or any country other than Germany where the LPHA assumed the case acquired the infection. In case of an uncertain place of infection, two or more counties or countries may be entered into the surveillance database. Therefore, by definition, the place of infection for NNO cases is the county where the hospital is located.

#### Hospitalisation

For a notified case to be reported as hospitalised, the national surveillance system does not require a causal link between the notified disease and hospitalisation. Therefore, by definition, for NNOs all patients are to be reported as hospitalised. Hospitalisation of hospital staff for norovirus infection is very unlikely and hospital staff infected during NNOs was assumed to be correctly reported as “not hospitalised”.

### Follow-up of norovirus laboratory notifications by the 19 Hessian LPHAs

To quantify the influence of the different follow-up procedures on the number of nosocomial norovirus outbreaks and cases captured in the surveillance system the 19 LPHAs were questioned regarding their follow-up procedures and then grouped based on whether they routinely contact all persons for whom a laboratory diagnosis of norovirus infection is notified (complete follow-up) or whether follow-up of laboratory notifications is limited to notifications suggesting an institutional setting (incomplete follow-up). For both groups of LPHAs, the number of hospital beds, the number of outbreaks per 1,000 hospital beds per year and the number of cases within NNOs for acute care and rehabilitation hospitals were calculated. The two groups of LPHAs were compared by calculating incidence-rate ratios (IRRs), 95% confidence intervals (95% CIs), and attributable fractions among the exposed and population attributable fractions [20]. Differences in outbreak sizes between the two groups were tested using the Wilcoxon rank-sum test.

### Time requirements to link outbreak-related cases at the Hessian state level

As part of the routine surveillance activities the SurvNet@RKI database at the Hessian SPHA was checked weekly for new or updated outbreak associated norovirus cases and outbreak cases linked to outbreak reports. In 2007, working-time required to check reported norovirus cases and link outbreak associated cases was prospectively documented. This included comparing OIN of newly received norovirus cases to previously received cases and, if applicable, combining them in the dataset to outbreak reports. The time required for Norovirus cases reported from all 25 Hessian counties was documented, as in practice it was not possible to separately determine working-time by county or outbreak. Time required for further aspects of the surveillance system e.g. for the collection, analysis and dissemination of data, or time required for feed-back to LPHAs of missing or erroneous information on OINs or outbreak setting, was not included. Nor could we document the time requirements at the local level.

### Population under surveillance in counties included in the evaluation

The evaluation was conducted for 19 counties in Hesse, Germany, with a total population of 4.2 million inhabitants. These were the 19 counties who’s LPHAs agreed to participate in the NNO study. 114 acute care and 45 rehabilitation hospitals operate in these 19 counties, providing 20,160 and 8,723 beds, respectively. The time required to link outbreak-related cases at the Hessian state level was documented for all 25 Hessian counties (6.1 million inhabitants). Population data and number of hospital beds by county were provided by the Hesse Statistical Office, Germany (Hessisches Statistisches Landesamt, Wiesbaden, Germany).

## Results

### Outbreak size

Information on 155 NNOs that began in 2007 was obtained through the national surveillance system and the NNO control study. However, for these 155 outbreaks, the surveillance dataset contained 3,115 cases and the NNO control study dataset 3,381 cases. For 86 (55%) of these outbreaks, outbreak size did not differ between the two datasets. For 58 (37%) outbreaks, the surveillance dataset contained fewer cases, and for 11 (7%) outbreaks the surveillance dataset contained more cases. For 65 (94%) of the 69 outbreaks with discrepant information on outbreak size, the differences could be clarified (table 1).

**Table 1:** Differences in outbreak size between the two datasets, nosocomial norovirus outbreaks, Hesse, Germany, 2007.

Reasons for differences in outbreak size	Number of outbreaks	Total number of cases
**More cases in the surveillance dataset (Number of outbreaks = 11)**		
LPHA included cases of a linked nursing home in hospital outbreak	1	22
No explanation	3	19
LPHA responsible for outbreak not informed on additional cases identified by 2^nd^ LPHA	2	13
LPHA miscounted cases on line list when filling out the questionnaire for the NNO control study	3	8
Cases assigned mistakenly to outbreak	2	2
**Less cases in the surveillance dataset (N = 58)**		
County of residence different from county of outbreak	53	367
only aggregated numbers reported to LPHA	1	21
No explanation	1	8
LPHA missed cases when entering data into the local database	1	2
LPHA miscounted cases on line list when filling out the questionnaire for the NNO control study	2	2

### Place of infection

In the surveillance dataset, information on the place of infection was available for 99.3% (3,094/3,115) of the Hessian NNO cases. Eight-hundred-thirty (27%) of the 3,315 cases were forwarded from the LPHA managing the outbreak to the LPHA responsible for the county of residence of a case. For 820 of these cases, one place of infection was reported. For 199 (24%) of the 820 cases, the place of infection reported was different from the outbreak county and mostly corresponded to the county of the LPHA entering the data.

### Hospitalisation

In the surveillance dataset, information on whether cases were hospitalised was available for 99.7% (3,105/3,115) of NNO cases. Thirty-four percent (1,052/3,105) of these cases were reported as hospitalised. In the NNO control study dataset, 81% (2,721) of persons affected were patients, and the remainder were staff members. Therefore, for an estimated 47% of Hessian NNO cases, information on hospitalisation was incorrect in the surveillance dataset.

### Follow-up of notifications by LPHAs

Fifteen of the 19 LPHAs routinely contacted all persons for whom a laboratory diagnosis of norovirus infection was notified. Number of outbreaks, number of hospital beds and number of outbreaks per 100 hospital beds for counties with and without complete follow-up are presented in table 2. Reported NNO incidence in acute care hospitals was higher in counties with complete follow-up of laboratory notifications than in counties with incomplete follow-up (IRR 2.7, 95% CI 1.4–5.7, p-value  = 0.002). For NNO in rehabilitation clinics this difference was small and did not reach statistical significance (IRR 1.4, 95% CI 0.4–8.0, p-value  = 0.6). Also, counties with complete follow-up of laboratory notifications reported in total more NNO-cases for acute care hospitals (IRR 2.1, 95% CI 1.9–2.4, p-value  = 0.001), but not for rehabilitation clinics (IRR 0.9, 95% CI 0.7–1.2, p-value  = 0.4). For acute care hospitals, we obtained an attributable fraction among the exposed of 63%, i.e. for counties with complete follow-up 63% of outbreaks in acute care hospitals were identified because LPHAS following-up all laboratory notifications instead of following-up only those suggesting an institutional setting. The population attributable fraction, i.e. the net proportion of all outbreaks known to the 19 LPHAs being identified because of 14 LPHAs completely following-up all laboratory notifications instead of following-up only laboratory notifications suggesting an institutional setting, was 59%. The 13 outbreaks in counties with incomplete follow-up were nearly twice as large as the 142 outbreaks in counties with complete follow-up (median 21 and 11 cases, respectively) (p-value  = 0.08).

**Table 2:** Number of outbreaks, number of hospital beds and number of outbreaks per 100 hospital beds for counties with and without complete follow-up of laboratory notifications, Hesse, Germany, 2007.

Completeness of follow-up	Number of outbreaks	Number of hospital beds	Incidence Rate (Number of outbreaks per 100 hospital beds)	Incidence Rate Ratio (95% CI)
**Acute care hospitals**				
Complete	131	16,725	7.8	2.7 (1.4–5.7)
Incomplete	10	3,435	2.9	Ref.
**Rehabilitation hospitals**				
Complete	11	6,281	1.8	1.4 (0.4–8.0)
Incomplete	3	2,442	1.3	Ref.

### Time requirements to link norovirus cases at the Hessian state level

For 2007, the surveillance database SurvNet@RKI contained 12,115 norovirus cases reported in Hesse. At the Hessian state level, working-time to link norovirus cases reported in 2007 was 151 hours.

## Discussion

In Europe, rates of ascertained viral gastroenteritis outbreaks differ markedly, suggesting incomplete ascertainment for most countries [9]. In our evaluation, LPHA with complete follow-up of laboratory notifications of norovirus infection identified more outbreaks and more outbreak associated cases than LPHAs who only contacted cases whose notifications suggested an institutional setting, i.e. a hospital laboratory. Calculation of the population attributable risk percent indicated that more than half of NNOs were identified because a part of the Hessian LPHAs followed-up all laboratory notifications, irrespective of their chance to find an outbreak. This calculation did not measure the total contribution of follow-up of laboratory notifications. Even LPHAs with incomplete follow-up identified NNO based on laboratory notifications. Our evaluation confirms previous, anecdotal reports of NNO being underreported in Germany and the contribution of LPHAs for ascertainment of NNO by actively searching for outbreaks. The tendency towards a higher median outbreak size in counties with incomplete follow-up also suggests a differential notification of outbreaks by size. Previously it had been hypothesized that surveillance databases for Norovirus outbreaks preferentially include larger outbreaks, as outbreaks are not reported until they reach a certain size [21].

The use of a second dataset – the NNO control study dataset – suggests that all NNOs known at Hessian LPHAs could be retrieved from the national surveillance database. Forwarding of outbreak cases by the LPHA managing the outbreak to the LPHAs responsible for the cases’ counties of residence was the major factor explaining the missing of notified cases in the surveillance dataset. For the 155 NNOs contained in the surveillance dataset (including 3,115 cases), 367 cases were missed for this reason, indicating that mean estimates of outbreak size would be biased by 2.4 cases per outbreak. For these missed cases we were unable to assess if they had not been entered into the surveillance system or if they had been entered without OIN enabling us to identify cases as outbreak-associated cases and to link them.

More than one quarter of all ascertained cases in NNOs needed to be forwarded to the LPHA responsible for the cases’ counties of residence. Forwarding of case-based information to a second LPHA for data entry implies additional work: 1) for the hospital to provide LPHAs with address details of cases, 2) for LPHAs to forward case-based information and 3) for state and national institutes to link reported cases. In our evaluation, only resources required to link cases at the Hessian state level were quantified. Under the assumption of these time requirements depending exclusively on population size, extrapolating the Hessian SPHA time requirements to all 16 German SPHAs (responsible for 82 million inhabitants), time requirements for 2007 would have amounted to 254 eight-hour working days. In our view, this effort is far too huge to be justified.

The purpose of forwarding case-based information to the LPHA responsible for the cases’ county of residence is to allow the LPHA to conduct further infection control investigations and prompt appropriate measures, such as at home or in the cases' workplace. However, for many nosocomial norovirus cases, no further infection control investigations or measures are required in the county of residence of the case and forwarding of the information does not lead to any infection control action by the LPHA receiving it. We therefore believe that the LPHA managing an institutional outbreak should enter directly all outbreak-related data into the surveillance system.

Besides the linking of norovirus cases at state level, additional factors influence reported NNO sizes: hospitals test different numbers of symptomatic patients for norovirus, tests used for norovirus diagnosis have different sensitivities and specificities [22], and distinguishing between (ongoing) transmission and repeated norovirus introduction into a hospital is difficult [23]. We therefore think that for mandatory surveillance, current efforts to produce “accurate” data on NNO sizes by linking of cases are disproportionate.

Completeness of data on hospitalisation and county of infection was high (over 99%). For county of infection, all software programs provide a default answer selection, corresponding to the county the LPHA is responsible for. This pre-selection explains the high completeness for this variable, but also the high proportion of incorrect entries (24%) when the pre-selection should have been changed. For the variable hospitalisation, no pre-selection is possible and the high proportion of incorrect entries (47%) is a result of LPHAs’ misconception of the variable’s meaning: when the results of this evaluation were fed-back to LPHAs’ staff, many of them reported to require a causal association, i.e. hospitalisation for norovirus infection, thus erroneously not reporting cases who acquired their infection during hospitalisation as hospitalised. The high proportion of incorrect data entries 1) generally questions the validity of the current surveillance data regarding the proportion of notified (norovirus) cases being hospitalised and 2) suggests reconsidering the surveillance definition of hospitalisation (e.g. into “yes, due to norovirus infection”; “yes, due to other causes”; “no”) and 3) underlines the need for data quality efforts to obtain useful case-based information from routine surveillance.

Norovirus-Infections are not on the list of priority diseases for surveillance in the European Union [24] and large differences in the surveillance systems for Norovirus-infections exist. E.G. France, Denmark, and Sweden report only suspected food-borne Norovirus outbreaks, Italy and Spain do not have a national Norovirus surveillance system [25], and in Austria all viral foodborne infections have to be notified [26]. Published reports on numbers of Norovirus outbreaks for Germany are very high when compared to other European countries [9], [25]. In Florida (>18 million inhabitants) only 7 out of 257 reported Norovirus outbreaks during a 1-year study affected hospitals [27]. To our knowledge, the German surveillance system is unique in combining a huge number of Norovirus outbreak reports with the availability of case-based information for all cases, whether outbreak associated or not, at the national level.

In addition to collecting case-based information on huge numbers of Norovirus cases, the work required to provide this information for all outbreak cases for the state and national level is increased by the current procedure of data entry of Norovirus cases belonging to one outbreak in several LPHAs and linking the information at state and national level. Furthermore, the extraction of setting-specific outbreak data of the national surveillance database is intricate [11], and the national database contains high numbers of incorrect entries (e.g. on hospitalisation of Norovirus cases). We believe that at state and national level aggregated data, including a limited number of variables per outbreak (e.g. date of onset of first and last case, number of cases, outbreak setting, foodborne origin), would provide sufficient information to describe the epidemiology of norovirus outbreaks. It has been previously suggested that for diseases with a high incidence and low severity case-by-case reporting should not be required at the national level [28]. At the local level complete follow- up of laboratory notifications allowed LPHAs to identify NNOs and to discuss control measures with hospitals.

For the 2009–2010 norovirus season the RKI changed the reporting requirements for LPHAs in order to reduce their workload. While reporting requirements of laboratory confirmed Norovirus cases remained unchanged, non laboratory-confirmed, outbreak-associated cases could be reported in an aggregated form [29]. Beginning in 2011, LPHAs have to report only laboratory confirmed Norovirus cases [30]. Further changes of the system, e.g. the forwarding of case-based information from the LPHA managing the outbreak to the LPHA responsible for the cases county of residence, will require a change in the German Protection against Infection Act (IfSG). In our view further work is needed to find a better balance between data requirements for descriptive epidemiology, required resources and data quality issues for the surveillance of NNOs.
